# Generative AI in healthcare: an implementation science informed translational path on application, integration and governance

**DOI:** 10.1186/s13012-024-01357-9

**Published:** 2024-03-15

**Authors:** Sandeep Reddy

**Affiliations:** Deakin School of Medicine, Waurn Ponds, Geelong, VIC 3215 Australia

**Keywords:** Generative artificial intelligence, Healthcare, Implementation science, Translation pathway

## Abstract

**Background:**

Artificial intelligence (AI), particularly generative AI, has emerged as a transformative tool in healthcare, with the potential to revolutionize clinical decision-making and improve health outcomes. Generative AI, capable of generating new data such as text and images, holds promise in enhancing patient care, revolutionizing disease diagnosis and expanding treatment options. However, the utility and impact of generative AI in healthcare remain poorly understood, with concerns around ethical and medico-legal implications, integration into healthcare service delivery and workforce utilisation. Also, there is not a clear pathway to implement and integrate generative AI in healthcare delivery.

**Methods:**

This article aims to provide a comprehensive overview of the use of generative AI in healthcare, focusing on the utility of the technology in healthcare and its translational application highlighting the need for careful planning, execution and management of expectations in adopting generative AI in clinical medicine. Key considerations include factors such as data privacy, security and the irreplaceable role of clinicians’ expertise. Frameworks like the technology acceptance model (TAM) and the Non-Adoption, Abandonment, Scale-up, Spread and Sustainability (NASSS) model are considered to promote responsible integration. These frameworks allow anticipating and proactively addressing barriers to adoption, facilitating stakeholder participation and responsibly transitioning care systems to harness generative AI’s potential.

**Results:**

Generative AI has the potential to transform healthcare through automated systems, enhanced clinical decision-making and democratization of expertise with diagnostic support tools providing timely, personalized suggestions. Generative AI applications across billing, diagnosis, treatment and research can also make healthcare delivery more efficient, equitable and effective. However, integration of generative AI necessitates meticulous change management and risk mitigation strategies. Technological capabilities alone cannot shift complex care ecosystems overnight; rather, structured adoption programs grounded in implementation science are imperative.

**Conclusions:**

It is strongly argued in this article that generative AI can usher in tremendous healthcare progress, if introduced responsibly. Strategic adoption based on implementation science, incremental deployment and balanced messaging around opportunities versus limitations helps promote safe, ethical generative AI integration. Extensive real-world piloting and iteration aligned to clinical priorities should drive development. With conscientious governance centred on human wellbeing over technological novelty, generative AI can enhance accessibility, affordability and quality of care. As these models continue advancing rapidly, ongoing reassessment and transparent communication around their strengths and weaknesses remain vital to restoring trust, realizing positive potential and, most importantly, improving patient outcomes.

Contributions to the literature

- Generative AI has the potential to revolutionize clinical decision-making and improve health outcomes, but its utility and impact in healthcare remain poorly understood.
- The article outlines the vast capacity of generative AI to revolutionize healthcare system operations, scientific investigation and patient care, contending that if applied conscientiously, generative AI could enhance medical care quality, fairness and efficiency.
- Though generative AI holds promise, successfully integrating it into the intricate healthcare system requires carefully planned approaches. The article provides methodical integration plans informed by implementation science principles, which are vital for gradually and effectively transforming complex care environments with new technologies over time.

## Background

Artificial intelligence (AI) has become an increasingly popular tool in a variety of fields, including healthcare, with the potential to transform clinical decision-making and improve health outcomes [1–3]. Generative AI is one area of AI that has gained attention recently for its ability to use machine learning algorithms to generate new data, such as text, images and music [4–7]. Generative AI is proving to be a change catalyst across various industries, and the healthcare sector is no exception [8]. With its remarkable ability to analyse extensive datasets and generate valuable insights, generative AI has emerged as a powerful tool in enhancing patient care [9], revolutionizing disease diagnosis [10] and expanding treatment options [11]. By harnessing the potential of this cutting-edge technology, healthcare professionals can now access unprecedented levels of accuracy, efficiency and innovation in their practices.

Despite the potential benefits, the utility and impact of generative AI in healthcare remain poorly understood [12, 13]. The application of generative AI in healthcare raises ethical and medico-legal concerns [14]. Moreover, it is unclear how generative AI applications can be integrated into healthcare service delivery and how the healthcare workforce can utilise them appropriately [15]. Furthermore, it is uncertain how far generative AI can improve patient outcomes and how this can be assessed. Finally, the value of generative AI beyond augmenting clinical and administrative tasks needs to be explored.

Realizing generative AI’s vast potential in healthcare requires translational approaches rooted in implementation science. Such approaches recognize technological progress alone will not revolutionize healthcare overnight [16, 17]. Real change requires carefully orchestrated sociotechnical transitions that put people first. Implementation science-based approaches provide generalizable roadmaps grounded in empirical evidence from prior health IT deployments [16]. As such, healthcare leaders pioneering generative AI integration would be well served in leveraging these models to reinforce patient safety, trust and impact [17]. To facilitate the appropriate incorporation and application of generative AI in healthcare, this article aims to provide an overview of the use of generative AI in healthcare followed by guidance on its translational application.

## Generative AI

Generative AI is a class of machine learning technology that learns to generate new data from training data [18, 19]. Generative models generate data that is similar to the original data. This can be useful in a variety of applications such as image and speech synthesis. Another unique capability is that they can be used to perform unsupervised learning, which means that they can learn from data without explicit labels [8]. This can be useful in situations where labelled data is scarce or expensive to obtain. Furthermore, generative AI models can generate synthetic data by learning the underlying data distributions from real data and then generating new data that is statistically similar to the real data. Generative models differ from other types of machine learning models in that they aim to endow machines with the ability to synthesise new entities [8]. They are designed to learn the underlying structure of a dataset and generate new samples that are like the original data. This contrasts with discriminative models, which are designed to learn the boundary between different classes of data. These models focus on tasks such as classification, regression or reinforcement learning, where the goal is to make predictions or take actions based on existing data. There are several categories of generative AI, as outlined in Table 1 [20–23].

**Table 1:** Generative AI models [20–23]

**Generative AI model**	**Description**	**Applications**
**Generative adversarial networks (GANs)**	GANs consist of 2 neural networks, a generator and a discriminator, that compete against each other. GANs are often used in image synthesis, super-resolution, style transfer, and more	Image synthesis, style transfer, face ageing, data augmentation, 3D object creation
**Variational autoencoders (VAEs)**	VAEs are a type of autoencoder which adds additional constraints to the encoding process, causing the network to generate continuous, structured representations. This makes them useful for tasks such as generating new images or other data points	Image generation, anomaly detection, image denoising, exploration of latent spaces, content generation in gaming
**Autoregressive models**	These models predict the next output in a sequence based on previous outputs. They have been used extensively in language modelling tasks (like text generation), as well as in generating music and even images	Text generation (e.g., GPT models), music composition, image generation (e.g., PixelRNN), time-series forecasting
**Flow-based models**	These models leverage the change of variables formula to model complex distributions. They are characterised by their ability to both generate new samples and perform efficient inference	High-quality image synthesis, speech and music modelling, density estimation, anomaly detection
**Energy-based models (EBMs)**	In EBMs, the aim is to learn an energy function that assigns low-energy values to data points from the data distribution and higher energies to other points. EBMs can be used for a wide range of applications, including image synthesis, denoising and in painting	Image synthesis and restoration, pattern recognition, unsupervised and semi-supervised learning, structured prediction
**Diffusion models**	These models gradually learn to construct data by reversing a diffusion process, which transforms data into a Gaussian distribution. They have shown remarkable results in generating high-quality, diverse samples	High-fidelity image generation (DALL-E2), audio synthesis, molecular structure generation

While there are several generative AI models, this article will mainly focus on two models, which are popular in the healthcare context: *generative adversarial networks* and *large language models*.

### Generative adversarial networks

Generative adversarial networks (GANs) differ from traditional generative modelling techniques in their approach to learning [24]. GANs use a game-theoretic framework with competing networks. GANs consist of two neural networks, a generator and a discriminator, that compete against each other. The generator creates fake data to pass to the discriminator. The discriminator then decides if the data it received is like the known, real data. Over time, the generator gets better at producing data that looks real, while the discriminator gets better at telling the difference between real and fake data. This adversarial training process allows GANs to learn representations in an unsupervised and semi-supervised fashion. In contrast, traditional generative modelling techniques often rely on explicit probabilistic models or variational inference methods.

Recent developments in GANs relating to representation learning include advancements in learning latent space representations [24]. These developments focus on improving the ability of GANs to transform vectors of generated noise into synthetic samples that resemble data from the training set. Some specific examples of recent developments in this area include GANs applied to image generation, semi-supervised learning, domain adaptation, generation controlled by attention and compression [5]. These advancements aim to enhance the representation learning capabilities of GANs and enable them to generate more realistic and diverse samples.

GANs have been used to generate realistic images [24]. These models can learn the underlying distribution of a dataset and generate new images that resemble the original data. This has applications in areas like computer graphics, art and entertainment. Moreover, GANs can be used to augment training data by generating synthetic samples. This can help in cases where the original dataset is small or imbalanced, improving the performance of machine learning models. Synthetic data, created by machine learning algorithms or neural networks, can retain the statistical relationships of real data while offering privacy protection. Synthetic data is also being considered for enhancing privacy.

### Large language models

Large language models (LLMs) are powerful AI models that have shown promise in various natural language processing (NLP) tasks [25]. In particular, the availability of OpenAI’s GPT-4 [26], Anthropic’s Claude [27] and Google’s PaLM2 [28] has significantly galvanised the progress of not just NLP but the field of AI in general, whereby commentators are discussing achievement of human-level performance by AI [10, 29]. LLMs like OpenAI’s GPT-4 are based on the autoregressive model. An autoregressive model is used to generate sequences, such as sentences in natural language, by predicting a next item based on previous ones [30]. The difference between LLMs and traditional language models lies in their capabilities and training methods [25]. LLMs, like GPT-4, utilise the Transformers architecture, which has proven to be effective for understanding the context of words in a sentence. A transformer uses a mechanism called ‘attention’ to weigh the importance of words when making predictions [31]. This mechanism allows the model to consider the entire history of a sentence, making it a powerful tool for sequence prediction tasks. LLMs are trained on a large corpus of text data. During training, the model learns to predict the next word in a sentence given the previous words. It does this by adjusting its internal parameters to minimise the difference between its predictions and the actual words that follow in the training data.

One of the key advantages of LLMs is their ability to perform many language processing tasks without the need for additional training data [32]. This is because they have already been trained on a vast corpus of text, allowing them to generate coherent and contextually relevant responses based on the input they receive. This makes them particularly useful as references or oracles for text summarization models. Text summarization is a complex task that involves understanding the main points of a piece of text and then condensing these points into a shorter form. LLMs can be used to generate summaries of text, which can then be used as a reference or ‘gold standard’ for other summarization models [25]. This can help to improve the performance of these models by providing them with high-quality summaries to learn from.

In addition to text summarizations, LLMs have also been used in a variety of other applications [15]. In the realm of text classification, LLMs can be used to automatically categorise pieces of text into predefined categories. This can be useful in a variety of applications, from spam detection in email filters to sentiment analysis in customer reviews. Finally, LLMs have been used for the automatic evaluation of attribution. This involves determining the source or author of a piece of text. For example, an LLM could be used to determine whether a particular tweet was written by a specific person, or to identify the author of an anonymous piece of writing.

It is important to note that while LLMs are powerful, they have limitations [15]. Because they generate sequences one component at a time, they are inherently sequential and cannot be parallelised. Moreover, they are causal, meaning that they can only use information from the past, not the future, when making predictions [33, 34]. They can struggle to capture long-range dependencies because of the vanishing gradient problem, although architectures like Transformers help mitigate this issue.

## Application of generative AI in healthcare

**Fig. 1:**
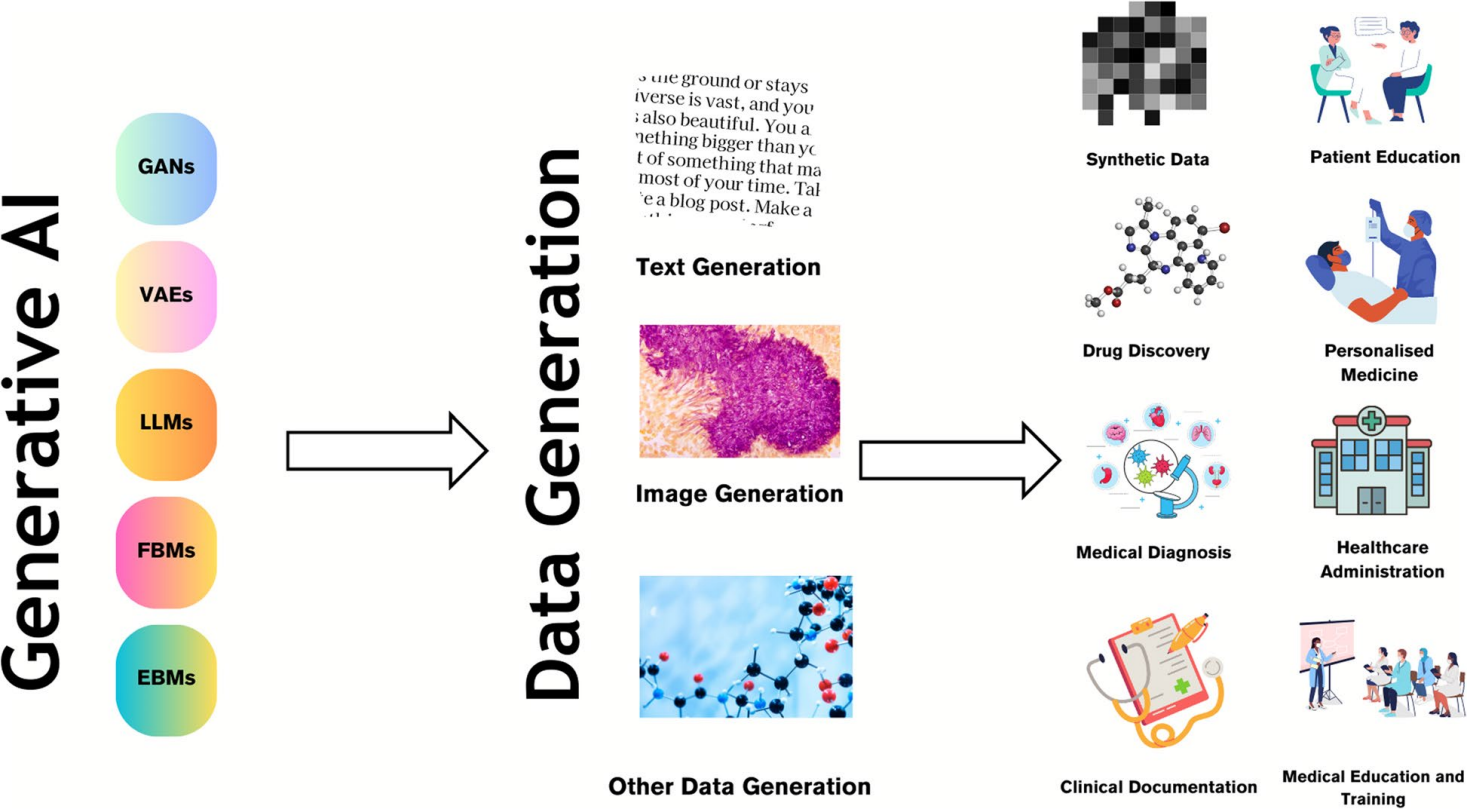
Use cases of generative AI in healthcare. Generative AI models like generative adversarial networks (GANs) and large language models (LLMs) are used to generate various data modalities including text and image data, which are then used for various scenarios including drug discovery, medical diagnosis, clinical documentation, patient education, personalized medicine, healthcare administration and medical education amongst other use cases

Generative AI models that facilitate the creation of text and images are seen as a promising tool in the healthcare context [26, 35, 36]. Generative AI can transform healthcare by enabling improvements in diagnosis, reducing the cost and time required to deliver healthcare and improving patient outcomes (Fig. 1).

### Synthetic data generation and data augmentation

Synthetic data, which is created using generative AI models like GANs, is an increasingly promising solution for balancing valuable data access and patient privacy protection [9]. By using generative AI models, realistic and anonymised patient data can be created for research and training purposes, while also enabling a wide range of versatile applications. Moreover, GANs can synthesise electronic health record (EHR) data by learning the underlying data distributions, which allows for excellent performance and addresses challenges such as data privacy concerns. This approach can be particularly useful in situations where there is a limited amount of real-world patient data available, or when access to such data is restricted due to privacy concerns. Additionally, the use of synthetic data can help to improve the accuracy and robustness of machine learning models, as it allows for a more diverse and representative range of data to be used in the training process. Furthermore, the ability to generate synthetic data with different characteristics and parameters can enable researchers and clinicians to investigate and test various hypotheses [5, 9, 37], leading to new insights and discoveries.

### Drug discovery

Generative AI models are also being used to generate novel small molecules, nucleic acid sequences and proteins with a desired structure or function, thus aiding in drug discovery [11]. By analysing the chemical structure of successful drugs and simulating variations, generative AI can produce potential drug candidates at a much faster rate than traditional drug discovery methods. This not only saves time and resources but can also help to identify drugs that may have gone unnoticed using traditional methods. Moreover, the use of generative AI can also aid in predicting the efficacy and safety of new drugs, which is a crucial step in the drug development process. By analysing vast amounts of data, generative AI can help to identify potential issues that may arise during clinical trials, which can ultimately reduce the time and cost of drug development [11, 38]. In addition, generative AI by identifying specific biological processes that play a role in disease can help to pinpoint new targets for drug development, which can ultimately lead to the development of more effective treatments.

### Medical diagnosis

Generative models can be trained on vast datasets of medical records and imagery (like MRIs and CT scans) to identify patterns related to diseases. For instance, GANs have been used for image reconstruction, synthesis, segmentation, registration and classification [5, 9, 37, 39]. Moreover, GANs can be used to generate synthetic medical images that can be used to train machine learning models for image-based diagnosis or augment medical datasets. LLMs can enhance the output of multiple CAD networks, such as diagnosis networks, lesion segmentation networks and report generation networks, by summarising and reorganizing the information presented in natural language text format. This can create a more user-friendly and understandable system for patients compared to conventional CAD systems.

EHRs and other patient records are rich repositories of data, and LLMs can be used to analyse these records in a sophisticated manner [40]. They can process and understand the information and terminology used in these records, which allows them to extract and interpret complex medical information. This capability extends beyond simple keyword matching, as LLMs can infer meaning from incomplete information, and even draw on a vast medical corpus to make sense of the data. Moreover, LLMs can integrate and analyse information from multiple sources within the EHR. They can correlate data from lab results, physician’s notes and medical imaging reports to generate a more holistic view of the patient’s health [10]. This can be particularly useful in complex cases where the patient has multiple conditions or symptoms that may be related.

LLMs, like GPT-4, have shown medical knowledge despite lacking medicine-specific training [10, 29]. One of the most impressive aspects of these models is their ability to apply this knowledge in decision-making tasks [10]. For example, when presented with a hypothetical patient scenario, an LLM can generate a list of potential diagnoses based on the symptoms described, suggest appropriate tests to confirm the diagnosis and even propose a treatment plan. In some studies, these models have shown near-passing performance on medical exams, demonstrating a level of understanding comparable to that of a medical student [29]. However, limits exist, and the models’ outputs may carry certain risks and cannot fully substitute outpatient physicians’ clinical judgement and decision-making abilities [14].

### Clinical documentation and healthcare administration

LLMs such as GPT-4 and PALM-2 can be used to generate summaries of patient data [41]. This could be particularly useful in healthcare settings where large amounts of data are collected and need to be interpreted quickly and accurately. For instance, an EHR may contain patient data such as medical history, medications, allergies and laboratory results. A generative AI model could be trained to read through this data, understand the key points and generate a concise summary. This summary could highlight critical information such as diagnosis, prescribed medications and recommended treatments. It could also identify trends in the patient’s health over time. By automating this process, healthcare providers could save time and ensure that nothing important is overlooked. Furthermore, these summaries could be used to improve communication between different healthcare providers and between providers and patients, as they provide a clear and concise overview of the patient’s health status. The ability of LLMs to automate such processes can alleviate the current documentation burden and the consequent burnout many physicians across the world face [41]. Currently, many clinicians, due to organisational policies or health insurance requirements, are required to fill in lengthy documentation beyond what is required for routine clinical care. Studies have shown that many physicians spend over 1 h of time on electronic health record tasks for every hour of direct clinical face time [42]. Additionally, the cognitive load and frustration associated with documentation can reduce work satisfaction. contributing to their burnout [43]. Implementation of natural language processing tools to automate documentation could lessen this burden. An LLM embedded in the relevant information platform can undertake the documentation and provide draft versions for the clinician to approve [40, 41]. For example, hospitals can use LLMs to generate routine progress notes and discharge summaries [44].

Further to this, there is potential for these LLM-based applications to reduce medical errors and capturing missed information by providing a layer of scrutiny when embedded in EHRs [45]. In addition to automating documentation, LLMs integrated into EHRs could help reduce medical errors and ensure important information is not missed. Studies have found that many hospital patients will experience a preventable medical error, often due to issues like misdiagnosis, prescription mistakes or examination findings that are not followed up correctly [46]. Also, LLMs have the potential to serve as a decision support tool by analysing patient charts and flagging discrepancies or gaps in care [45]. For example, an LLM could cross-reference current symptoms and diagnostics against past medical history to prompt physicians about conditions that require further investigation. Additionally, they could scan medication lists and warn of potential adverse interactions or contraindications.

Generative AI can also be used to automate routine tasks in healthcare, such as scheduling appointments, processing claims and managing patient records [47]. For example, AI models can be used to develop intelligent scheduling systems. These systems can interact with patients through chatbots or voice assistants to schedule, reschedule or cancel appointments. They can consider factors such as doctor’s availability, patient’s preferred time and urgency of the appointment to optimize the scheduling process. Generative AI can also automate the process of insurance claims. It can read and understand the claim documents, verify the information, check for any discrepancies and process the claim. This can significantly reduce the time taken to process claims and minimise errors. By automating these tasks, healthcare providers can save time and resources and improve the patient experience as they get faster responses and more efficient service.

### Personalized medicine

Generative AI can analyse a patient’s genetic makeup, lifestyle and medical history to predict how they might respond to different treatments [48]. This is achieved by training the AI on large datasets of patient information, allowing it to identify patterns and correlations that might not be immediately apparent to human doctors. For example, the AI might notice that patients with a certain genetic marker respond particularly well to a specific medication. This information can then be used to create a personalized treatment plan that is tailored to the individual patient’s needs. This approach can lead to more effective treatment, as it considers the unique factors that might affect a patient’s response to medication. It can also lead to improved patient outcomes, as treatments can be optimized based on the AI’s predictions [48].

Generative AI can also be utilised in the field of mental health, particularly in the creation of interactive tools for cognitive behavioural therapy (CBT) [49, 50]. CBT is a type of psychotherapy that helps patients manage their conditions by changing the way they think and behave. Generative AI can be used to create personalized scenarios and responses that are tailored to the individual patient’s needs. For example, the AI might generate a scenario that triggers a patient’s anxiety, and then guide the patient through a series of responses to help them manage their reaction. This can provide patients with a safe and controlled environment in which to practice their coping strategies, potentially leading to improved mental health outcomes.

### Medical education and training

In the context of medical education and training, this technology can be used to generate a wide variety of virtual patient cases. These cases can be based on a diverse range of medical conditions, patient demographics and clinical scenarios, providing a comprehensive learning platform for medical students and healthcare professionals [51, 52]. One of the primary benefits of using generative AI in medical education is the ability to create a safe and controlled learning environment. Medical students can interact with these virtual patients, make diagnoses and propose treatment plans without any risk to real patients. This allows students to make mistakes and learn from them in a low stake setting. Generative AI can also create patient cases that are rare or complex, giving students the opportunity to gain experience and knowledge in areas they might not encounter frequently in their clinical practice. This can be particularly beneficial in preparing students for unexpected situations and enhancing their problem-solving skills. Furthermore, the use of AI in medical education can provide a more personalized learning experience. The AI can adapt to the learning pace and style of each individual, presenting cases that are more relevant to their learning needs. For example, if a student is struggling with a particular medical condition, the AI can generate more cases related to that condition for additional practice.

In addition to creating virtual patient cases, generative AI can also be used to simulate conversations between healthcare professionals and patients [51, 52]. This can help students improve their communication skills and learn how to deliver difficult news in a sensitive and empathetic manner. Moreover, the integration of AI in medical education can provide valuable data for educators. The AI can track the performance of students, identify areas of improvement and provide feedback, helping educators to refine their teaching strategies and curricula.

### Patient education

Generative AI can be used for patient education in several ways [35, 41]. It can be used to create personalized educational content based on a patient’s specific condition, symptoms or questions. For example, if a patient has diabetes, the AI can generate information about managing blood sugar levels, diet, exercise and medication. Generative AI can also engage patients in interactive learning experiences. Patients can ask questions, and the AI can generate responses, creating a dialogue that helps the patient understand their condition better. This can be particularly useful for patients who may be shy or embarrassed to ask certain questions to their healthcare providers. Furthermore, generative AI can also create visual aids, such as diagrams or infographics, to help patients understand complex medical concepts. For example, it could generate a diagram showing how a particular drug works in the body.

Generative AI can be programmed to generate content at different reading levels, helping to improve health literacy amongst patients with varying levels of education and comprehension [53]. It can also be used to create follow-up educational content and reminders for patients. For example, it could generate a series of emails or text messages reminding a patient to take their medication, along with information about why it is important. In addition, generative AI can be used to provide mental health support, generating responses to patients’ concerns or anxieties about their health conditions. This can help patients feel more supported and less alone in their health journey. Finally, generative AI can generate educational content in multiple languages, making healthcare information more accessible to patients who do not speak English as their first language.

## Translational path

The translational path of generative AI in healthcare is a journey that involves the integration of this advanced technology into the clinical setting [54]. This process has the potential to revolutionize the way healthcare is delivered, by automating tasks and generating relevant information, thus enhancing the efficiency of healthcare delivery [26, 35]. Generative AI can automate routine tasks such as data entry, appointment scheduling and even some aspects of patient care like monitoring vital signs or administering medication. This automation can free up a significant amount of time for clinicians, allowing them to focus more on direct patient care. By reducing the administrative burden on healthcare providers, generative AI can help improve the quality of care and increase patient satisfaction [41, 53]. In addition to automating tasks, generative AI can also generate relevant information for clinicians. For example, it can analyse patient data to predict health outcomes, identify potential health risks and suggest personalized treatment plans. This ability to generate insights from data can help clinicians make more informed decisions about patient care, potentially leading to improved patient outcomes.

However, the accuracy and completeness of the information generated by AI are crucial. Inaccurate or incomplete information can lead to misdiagnosis or inappropriate treatment, which can harm patients [14, 55]. Therefore, it is essential to ensure that the AI systems are well designed and thoroughly tested to produce reliable results. Despite the potential benefits, adopting generative AI in clinical medicine is not a straightforward process. It requires careful planning and execution [56]. This includes understanding the needs of the healthcare providers and patients, selecting the right AI technology, integrating it into the existing healthcare systems and training the staff to use it effectively. Moreover, there are also legal and ethical considerations, such as data privacy and security, that need to be addressed. Furthermore, it is important to manage expectations about what generative AI can and cannot do. Clinicians’ expertise and their ability to empathize with patients are still crucial in providing high-quality care.

The successful translation of generative AI into clinical practice hinges on thoughtful adoption strategies grounded in implementation science. Two models offer robust scaffolds: the technology acceptance model (TAM) at the individual user level [57] and the Non-Adoption, Abandonment, Scale-up, Spread and Sustainability (NASSS) framework for organisational integration [58]. Grounded in sociopsychological theory, TAM provides an evidence-based model for how end-user perceptions shape acceptance of new technologies like generative AI [59]. Its core tenets posit that perceived usefulness and perceived ease of use prove most determinative of uptake. TAM offers a quantifiable approach for predicting and influencing adoption that deployment efforts must consider. Segmenting staff and assessing beliefs allows tailored interventions addressing barriers like skills gaps, engagement, workflow integration and demonstrable benefits. Equally crucial, the NASSS framework delivers a holistic methodology assessing multi-level variables implicated in successfully embedding innovations. Its seven critical domains encompass technology design, value propositions, adopter priorities, organisational dynamics, wider contextual factors and their complex interplay [58]. Together, these lenses reinforce introduced generative AI responsibly, monitor progress and recalibrate based on emerging feedback. Melding TAM and NASSS perspectives provides a powerful blueprint for thoughtfully ushering generative AI into the twenty-first-century healthcare. They bring implementable strategies for the sociotechnical transition such innovations necessitate, promoting buy-in, facilitating integration, delivering sustained value and ultimately transforming care.

Based on these frameworks, the below content discusses the key components or steps a healthcare organisation or service should consider in integrating generative AI in their service delivery. The description will enable partners as to how to prepare their organisations and workforce to adopt and integrate generative AI to enable optimal care delivery. However, the description does not cover wider policy and legislative aspects that are required to facilitate the introduction of generative AI to healthcare. These characteristics are unique to various jurisdictions and continue to evolve rapidly, therefore are considered beyond the scope of this article.

### First component: acceptance and adoption

The successful implementation of AI in healthcare hinges on the understanding and acceptance of its applications by end users [54], including medical professionals and patients. This comprehension fosters trust in AI systems, enables their effective use and aids in navigating ethical and regulatory challenges. Moreover, a solid grasp of AI promotes continuous learning and adaptation to the evolving landscape of AI technology. Therefore, investment in improving awareness for all partners is crucial to ensure the effective adoption and utilisation of AI in healthcare.

Utilising the TAM and NASSS frameworks to the implementation generative AI in healthcare involves consideration of the following components:

- *▪ Perceived usefulness*: This refers to the degree to which a person believes that using a particular system would enhance his or her job performance. In the context of generative AI in healthcare, this could be how the AI can help in diagnosing diseases, predicting patient outcomes, personalizing treatment plans and improving administrative efficiency. For instance, AI could generate predictive models for patient outcomes based on their medical history, current health status and a vast database of similar cases.
- *▪ Perceived ease of use*: This refers to the degree to which a person believes that using a particular system would be free of effort. For generative AI in healthcare, this could mean how easy it is for healthcare professionals to understand and use the AI system. This includes the user interface, the clarity of the AI’s outputs and the level of technical support available.
- *▪ Attitude towards using*: The value proposition of generative AI in healthcare is compelling, offering benefits like cost-effectiveness, speed and personalized treatment options [5]. If healthcare professionals perceive the AI system as useful and easy to use, they are likely to develop a positive attitude towards using it. This positive attitude could be further enhanced by providing adequate training and support and by demonstrating the successful use of AI in similar healthcare settings.
- *▪ Behavioural intention to use*: Once healthcare professionals have a positive attitude towards the AI system, they are more likely to intend to use it. This intention could be turned into actual use by providing opportunities to use the AI system in a safe and supportive environment and by integrating the AI system into existing workflows.
- *▪ Actual system use*: The final step is the actual use of the AI system in daily healthcare practice. This could be encouraged by providing ongoing support and by continuously monitoring and improving the AI system based on user feedback and performance data.

In addition to these factors, the model also suggests that external factors like social influence and facilitating conditions can influence the acceptance and use of a new technology [57, 59]. In the case of generative AI in healthcare, these could include regulatory approval, ethical considerations, patient acceptance and the overall healthcare policy and economic environment.

### Second component: data and resources

Adopting generative AI involves preparing data and resources within an organisation to effectively utilise this technology. This is a complex process requiring a systematic and strategic approach that involves several key steps.

- *▪ Identifying use cases*: Healthcare organisations need to begin by identifying the specific use cases where generative AI can bring value. Generative AI aims to address various medical conditions, from chronic diseases like diabetes to acute conditions like stroke [6, 38, 60]. The complexity of the medical condition often dictates the level of sophistication required from the AI model. For instance, using AI for diagnostic imaging in cancer is complex and requires high accuracy. Understanding the specific use cases will help guide the data preparation process.
- *▪ Data collection*: Generative AI models learn from data [8], so the healthcare organisation needs to collect and prepare relevant data for training the models. This could involve gathering existing primary data from various sources within the organisation or collecting new data if necessary. The data then needs to be cleaned and preprocessed, which may involve tasks such as removing duplicates, handling missing values and normalizing data.
- *▪ Data cleaning and preprocessing*: It is necessary to clean and preprocess the collected data to ensure its quality and consistency [61, 62]. This may involve removing duplicates, handling missing values, standardizing formats and addressing any other data quality issues. Preprocessing steps may also include data normalization, feature scaling and data augmentation techniques to enhance the training process. It is important to highlight the need for uniformity in the quality of the datasets to enable seamless cross-functional data integration. Also, data quality is crucial as generative AI algorithms learn from data. The quality of data can be affected by various factors such as noise, missing values, outliers, biased data, lack of balance in distribution, inconsistency, redundancy, heterogeneity, data duplication and integration.
- *▪ Data annotation and labelling*: Depending on the use case, the organisation may need to annotate and label the data to provide ground truth and clinical standard information for training the generative AI models, specifically for fine-tuning LLMs with local data [10]. This could involve tasks such as image segmentation, object detection, sentiment analysis or text categorization. Accurate and comprehensive annotations are essential for training models effectively.
- *▪ Data storage and management*: Their will be a requirement to establish or utilise a robust data storage and management system to handle the large volumes of data required for generative AI. This may involve setting up a data warehouse, cloud storage or utilising data management platforms. All the while ensuring that the data is organised, accessible and secure for efficient training and model deployment. Data federation is a technology that can be considered here as it enables the creation of a physically decentralized but functionally unified database. This technology is particularly useful in healthcare as it allows various sources of data to keep the data within their firewalls. However, this step may not be required in most instances of the use of LLMs, particularly if they are drawn upon through application programming interface (API) calls or cloud services.
- *▪ Computational resources*: Generative AI models often require significant computational power and resources for training and inference such as GPUs or cloud computing services [8, 15]. In-house development and training of LLMs requires significant computational resources, which organisations must carefully consider [63]. Commercial LLMs offered through cloud services or APIs spare organisations this infrastructure burden. However, for those intent on training proprietary models tuned to their specific data and use cases, securing sufficient computing capacity is critical.

Factors that impact computational requirements include model size, training data volume and speed of iteration desired. For example, a firm aiming to train a model with over a billion parameters on tens of billions of text examples would likely pursue a high-performance computing cluster or leverage cloud–based machine learning platforms. The precise hardware configuration—including GPUs/TPUs, CPUs, memory, storage and networking—scales with the model architecture and training plan [63].

Ongoing model development and fine-tuning also necessitates available compute. Organisations can choose between continuing to allocate internal resources or outsourcing cycles via cloud services [63]. Budgetary planning should account for these recurring compute demands if continually enhancing in-house LLMs is a priority. Overall, while leveraging external LLMs can minimise infrastructure investments, serious internal LLM initiatives can rival the computational scale of industrial research labs.

### Third component: technical integration

Integrating generative AI into a healthcare information system or platform can bring numerous benefits, such as improved disease diagnosis, enhanced patient monitoring and more efficient healthcare delivery. However, generative AI technologies like GANs and LLMs are complex to understand and implement [8]. The technology’s maturity, reliability and ease of integration into existing systems are crucial factors affecting its adoption [58]. Therefore, integrating generative AI into a hospital or healthcare information system involves several steps ranging from understanding the needs of the system to implementing and maintaining the AI solution. The first step in integrating generative AI into a healthcare system is to identify the focus area of implementation [62]. This could be anything from improving patient care, streamlining administrative tasks, enhancing diagnostic accuracy or predicting patient outcomes. Once the need is identified, the right AI model needs to be chosen. Generative AI models, such as GANs, can be used for tasks like synthesising medical images or generating patient data [6, 37). LLMs can be used for EHR analysis and as a clinical decision support tool [40]. Once the model is chosen, it needs to be trained on the collected data. This involves feeding the data into the model and adjusting the model’s parameters until it can accurately predict outcomes or generate useful outputs.

Once the AI model is trained and tested, it can be integrated into the healthcare information system [56, 62]. This involves developing an interface between the AI model and the existing system, ensuring that the model can access the data it needs and that its outputs can be used by the system. Developing such an interface or API allows the generative AI models to be seamlessly integrated into the organisational or clinical workflow. After integration, the AI system needs to be extensively tested to ensure its functionality, usability and reliability.

Regular maintenance is also necessary to update the model as new data becomes available and to retrain it if its performance drops [56, 62]. Furthermore, gathering regular/scheduled feedback from healthcare professionals will ensure the organisation can make necessary refinements to improve the system’s performance.

When leveraging external LLMs for healthcare applications, stringent data governance practices are imperative to safeguard sensitive patient information [64]. As text or speech data gets routed to third-party LLM services for analysis, the contents contain protected health information (PHI) and personally identifiable information (PII) that must remain confidential.

While LLMs themselves are static analysis models rather than continuously learning systems, the vendors hosting these models and powering predictions still physically or computationally access submitted data [65, 66]. Irrespective of the vendors’ reassurances about privacy commitments, obligations and restrictions on ingesting customer content for model retraining, residual risks of data leakage or unintended retention persist. To mitigate these risks, comprehensive legal contracts between the healthcare organisation and LLM vendor are foundational to ensuring PHI/PII protection in accordance with health regulations. Business associate agreements, data usage agreements and master service provider contracts allow formally codifying allowable LLM data processing, storage, transmission and disposal protocols. Such contracts also establish liability and enforcement mechanisms in case of a breach attributed to the vendor, including notification, indemnification and restitution clauses. Strict access controls, encryption schemes, activity audit protocols and authorization procedures should complement these contractual protections. While LLMs themselves may not endlessly accumulate healthcare data like perpetually learning systems, due diligence around the long-term fate of data sent to LLM prediction services remains highly advisable for risk-averse legal and compliance teams [14]. Establishing robust data governance for emerging clinical LLM integration can prevent problematic regulatory, ethical and reputational exposure [64].

While beyond the scope of this article to discuss in detail, the organisation will additionally have a responsibility to ensure the AI system complies with relevant healthcare regulations and privacy laws [55], such as Health Insurance Portability and Accountability Act (HIPAA) in the USA or General Data Protection Regulation (GDPR) in the European Union.

### Fourth component: governance

While generative AI has several potential applications in clinical medicine, there are also several challenges associated with its implementation. Some of the challenges include the following:

- *▪ Data availability*: Generative AI requires large amounts of data to train models effectively [8]. However, in clinical medicine, data is often limited due to privacy concerns and regulations. This can make it difficult to train models effectively.
- *▪ Bias in training data*: Generative AI models require large amounts of training data to learn patterns and generate new data. If the training data is biased, the generative AI model will also be biased [13]. For example, if the training data is skewed towards a particular demographic group, the generative AI model may produce biased results for that group.
- *▪ Transparency*: While powerful LLMs like ChatGPT demonstrate impressive conversational ability, the opaque sourcing of their massive training corpora has rightly drawn scrutiny [64, 65]. Absent transparency around the origin, copyright status and consent policies of underlying data, legal and ethical blind spots remain. For commercially offered LLMs, details of training processes understandably remain proprietary intellectual property. However, the use of scraped web pages, private discussions, or copyrighted content without permission during model development can still create liability. Recent lawsuits alleging unauthorised scraping by LLM providers exemplify the growing backlash.
- *▪ Model interpretability*: Generative AI models can be complex and difficult to interpret, making it challenging for clinicians to understand how the model arrived at its conclusions [13, 67]. This can make it difficult to trust the model’s output and incorporate it into clinical decision-making.
- *▪ Inaccurate generation*: While LLMs demonstrate impressive fluency and versatility in conversational applications, their reliability breaks down when applied to high-stakes domains like healthcare [14, 55]. Without the contextual grounding in factual knowledge and reasoning capacity needed for medical decision-making, LLMs pose substantial patient safety risks if overly trusted by clinicians [14]. Hallucination errors represent one demonstrated failure mode, where LLMs confidently generate plausible-sounding but entirely fabricated responses lied outside their training distributions. For patient assessments, treatment plans or other clinical support functions, such creative falsehoods could readily culminate in patient harm if not rigorously validated [64]. Additionally, LLMs often ignore nuanced dependencies in multi-step reasoning that underlie sound medical judgments. Their capabilities centre on statistical associations rather than causal implications [68]. As such, they frequently oversimplify the complex decision chains requiring domain expertise that clinicians must weigh. Blindly accepting an LLM-generated diagnostic or therapeutic suggestion without scepticism can thus propagate errors.
- *▪ Regulatory and ethical issues*: The use of generative AI in clinical medicine raises several regulatory and ethical issues [14], including patient privacy, data ownership and accountability. Regulatory policies, ethical considerations and public opinion form the wider context. Data privacy laws like GDPR in Europe or HIPAA in the USA have implications for AI in healthcare [65]. These aspects need to be addressed to ensure that the use of generative AI is ethical and legal.
- *▪ Validation*: Generative AI models need to be validated to ensure that they are accurate and reliable [62]. This requires large datasets and rigorous testing, which can be time-consuming and expensive.

To minimise risks arising from the application of generative AI in healthcare, it is important to establish a governance and evaluation framework grounded in implementation science [64]. Frameworks such as the NASSS framework and the TAM should inform subsequent steps to promote responsible and ethical use of generative AI [58, 69]. This implementation science informed approach includes several steps to ensure appropriate testing, monitoring and iteration of the technology. The NASSS framework provides a useful lens for assessing the complex adaptive systems into which generative AI solutions would be embedded [58]. This framework examines factors like the condition, technology, value proposition, adopter system, organisation, wider context, and interaction and mutual adaptation over time. Analysing these elements can reveal barriers and enablers to adopting generative AI across healthcare organisations. Similarly, the TAM focuses specifically on human and social factors influencing technology uptake [59]. By evaluating perceived usefulness and perceived ease of use of generative AI systems, TAM provides insights into how both patients and providers may respond to and interact with the technology. TAM encourages stakeholder participation in system design to optimize user acceptance.

Both NASSS and TAM demand a thoughtful change management strategy for introducing new technologies like generative AI. This means conducting iterative testing and piloting of systems, co-developing governance policies with diverse voices, emphasizing transparency, providing extensive user training resources, developing protocols to assess AI quality and fairness, allowing user customization of tools, and continually evaluating impact to enable appropriate adaptation over time. Drawing from these models ensures responsible and ethical integration guided by end-user needs. The following are corresponding steps:

- *▪ Establish or utilise a governance committee*: This committee should be composed of experts in AI, healthcare, ethics, law and patient advocacy. The committee’s responsibility is to supervise the creation and implementation of generative AI applications in healthcare, making sure they adhere to the highest moral, statutory and professional standards.
- *▪ Develop relevant policies and guidelines*: Create policies and guidelines that address issues like data protection and security, informed consent, openness, responsibility and fairness in relation to the usage of generative AI in healthcare. The guidelines should also cover potential AI abuse and lay out precise reporting and resolution processes.
- *▪ Implement robust data management practices*: This includes ensuring data privacy and security, obtaining informed consent for data use, and ensuring data quality and integrity. It also involves using diverse and representative datasets to avoid bias in AI outputs.
- *▪ Mitigate inaccurate generated data*: Overall, while LLMs have strengths in certain narrow applications, their limitations in recalling latest findings, grounding advice in biomedical knowledge and deliberative analytical thinking pose risks in clinical roles [14]. Mitigating these requires both technological and process safeguards. At minimum, meticulous testing on massive, validated datasets, transparent uncertainty quantification, multi-modal human-AI collaboration and consistent expert oversight prove essential before contemplating LLM adoption for patient-impacting functions. With careful governance, LLMs may aid clinicians but cannot replace them.
- *▪ Risk assessment*: Prior to implementation, healthcare organisations must undertake structured risk assessments to inventory and quantify potential patient harms from generative AI adoption. Multi-disciplinary teams including clinicians, IT security, legal/compliance, risk management and AI engineers should participate. A broad examination of use cases, data dependencies, performance assumptions, safeguards, governance and liability scenarios provide the foundation. Identified dangers span clinical inaccuracies like inappropriate treatment suggestions to operational risks like biased outputs or diagnostics halted by technical outages. Other key considerations are malicious misuse, defects propagating as training data and breach of sensitive records compromising privacy or trust.

For each plausible risk, the assessment calibrates probability and severity estimates for variables like user types, information classes and mitigating controls. Continuous risk monitoring based on leading indicators and usage audits ensures the initial assessment adapts alongside inevitable model and application changes over time. Periodic probabilistic modelling using safety assurance methodologies further reinforces responsible governance. Overall, a nimble quantified risk approach prepares organisations to responsibly pursue generative AI’s benefits while protecting patients.

**Fig. 2:**
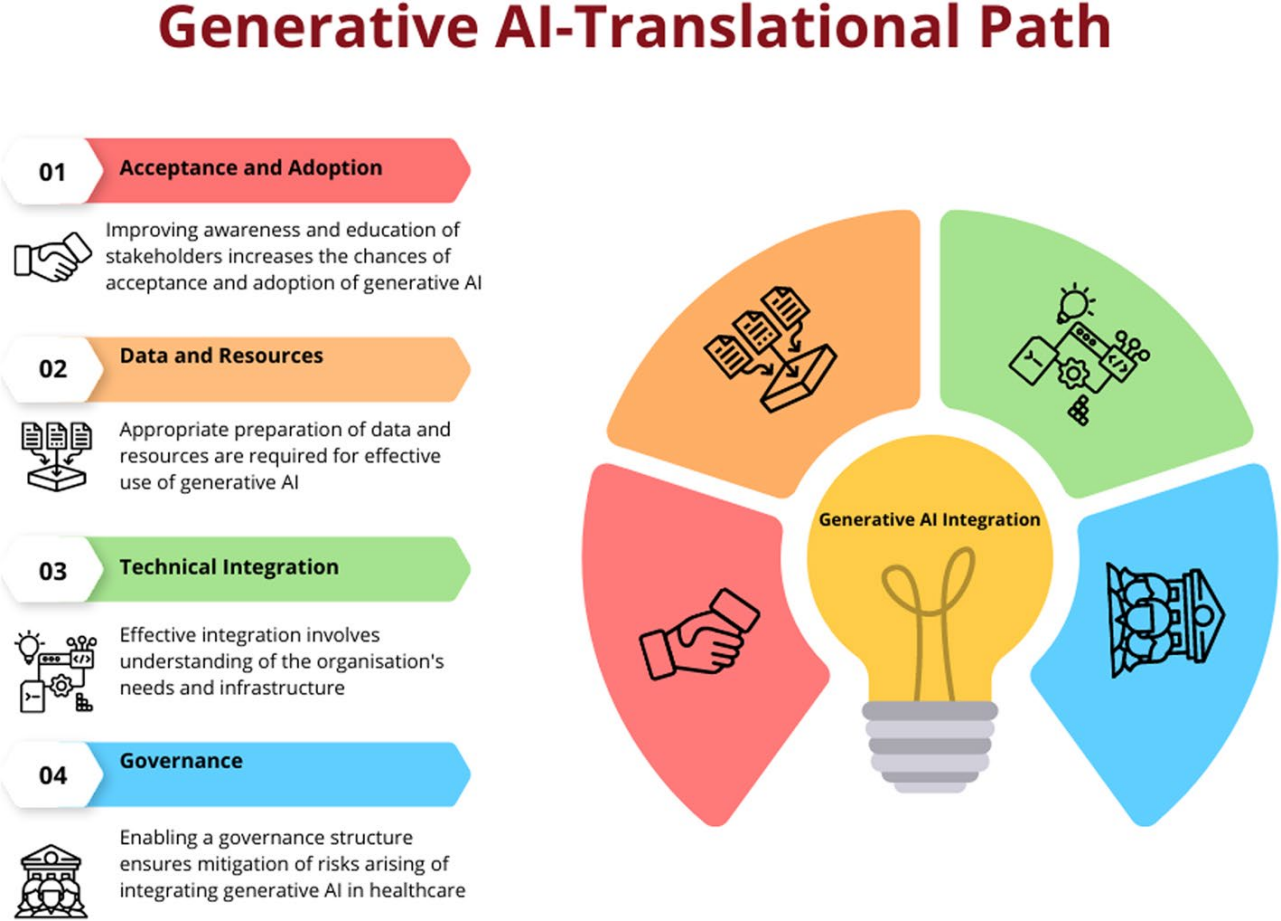
Translational path for generative AI in healthcare. Generative AI needs careful planning to incorporate it into healthcare delivery. Appropriate steps including ensuring there is acceptance amongst partners followed by planning for data acquisition and computation resources. Then after, integration and utilisation of generative AI in healthcare information systems is governed by a risk mitigation framework

- *▪ Ensuring transparency*: Ensure transparency of generative AI models by providing clear documentation of the underlying algorithms, data sources and decision-making processes. This promotes trust and enables healthcare professionals to understand and interpret the generated outputs. For risk-averse healthcare organisations, partnering with LLM vendors who refuse reasonable data transparency raises concerns. If unsuitable, illegal or fraudulent data underpins model predictions, patient safety and organisational reputation may suffer [13, 68]. Furthermore, litigation alleging regulatory noncompliance, privacy violations or misrepresentation based on questionable LLM data sourcing could follow [14]. Nonetheless, for many clinical functions, externally developed LLMs can sufficiently assist physicians without full transparency into underlying corpora. Simple conversational applications likely pose little concern. However, for more impactful care recommendations or patient-specific outputs, clinicians should validate suggestions accordingly rather than presume integrity [64]. Overall, the inaccessible nature of commercial LLM training data is an obstacle, but not a wholesale deal-breaker with careful governance around how predictions get utilised. Still, transparency remains an ongoing advocacy issue that healthcare providers should champion [64].
- *▪ Regulatory compliance*: Ensure compliance with relevant regulatory frameworks, such as data protection laws and medical device regulations. Collaborate with regulatory authorities to establish guidelines specific to generative AI in healthcare Establishing procedures for ongoing monitoring and evaluation of the models is crucial in addition to the measures to enable governance for the generative AI models [67, 70]. This involves collecting input from patients and healthcare experts as well as regular monitoring of performance, safety and ethical considerations. Healthcare organisations can reduce risks and guarantee the appropriate and ethical use of generative AI in healthcare by adhering to every step in this framework (Fig. 2). The governance framework harnesses the potential advantages of generative AI technology while promoting openness, responsibility and patient safety.

## Conclusion

Healthcare systems worldwide face crises of affordability, access and inconsistent quality that now endanger public health [71]. Generative AI presents solutions to begin rectifying these systemic failures through responsible implementation guided by scientific best practices.

Validated frameworks like the TAM and NASSS model provide actionable roadmaps for change management, stakeholder alignment and impact optimization [58, 59]. They allow anticipating adoption barriers related to perceived value, usability, risks and more while delineating interventions to drive acceptance. With meticulous planning grounded in evidence, generative AI can transform productivity, insight and care enhancement. Use cases like workflow and documentation automation, personalized predictive analytics, and patient education chatbots confirm vast potential [26, 41, 45], provided the technology supports rather than supplants human expertise. Structured integrations emphasizing clinician control safeguard quality while unlocking efficiency. Thoughtful translation is essential, but implementation science provides proven guidance.

The time for debate has passed. Patients worldwide stand to benefit, and responsible leaders must act urgently. Strategic pilots, iterative scaling and governance emphasizing ethics alongside innovation will realize long-overdue progress. Generative AI cannot single-handedly fix broken systems, but carefully facilitated adoption can catalyse reform while upholding healthcare’s humanitarian obligations. The approach, not just technology, defines success. Guided by wisdom and compassion, generative AI may help restore healthcare ideals so many now lack: quality, affordability and humane care for all.

## Data Availability

Data sharing is not applicable to this article as no datasets were generated or analysed during the current study.
